# Improved antimicrobial activities of *Boswellia sacra* essential oils nanoencapsulated into hydroxypropyl-beta-cyclodextrins†

**DOI:** 10.1039/d3na00882g

**Published:** 2023-12-22

**Authors:** Obaydah Abd Alkader Alabrahim, Salim Alwahibi, Hassan Mohamed El-Said Azzazy

**Affiliations:** a Department of Chemistry, School of Sciences & Engineering, The American University in Cairo AUC Avenue, SSE # 1184, P.O. Box 74 New Cairo 11835 Egypt Obaydah.alabrahim@aucegypt.edu hazzazy@aucegypt.edu +20 02 2615 2559; b Falha Medical Solutions Muscat 113 Oman; c Department of Nanobiophotonics, Leibniz Institute of Photonic Technology Jena Germany

## Abstract

Natural antimicrobials have recently gained increasing interest over synthetic antimicrobials to overcome foodborne pathogens and food microbial contamination. Essential oils (EOs) obtained from *Boswellia sacra* resins (BO) were utilized for respiratory disorders, rheumatoid arthritis, malignant tumors, and viral infections. Like other EOs, the therapeutic potential of BO is hindered by its low solubility and bioavailability, poor stability, and high volatility. Several studies have shown excellent physicochemical properties and outstanding therapeutic capabilities of EOs encapsulated into various nanocarriers. This study extracted BO from *B. sacra* resins *via* hydrodistillation and encapsulated it into hydroxypropyl-beta-cyclodextrins (HPβCD) using the freeze-drying method. The developed inclusion complexes of BO (BO-ICs) had high encapsulation efficiency (96.79 ± 1.17%) and a polydispersity index of 0.1045 ± 0.0006. BO-ICs showed presumably spherical vesicles (38.5 to 59.9 nm) forming multiple agglomerations (136.9 to 336.8 nm), as determined by UHR-TEM. Also, the formation and stability of BO-ICs were investigated using DSC, FTIR, FE-SEM, UHR-TEM, ^1^H NMR, and 2D HNMR (NOESY). BO-ICs showed greater thermal stability (362.7 °C). Moreover, compared to free BO, a remarkable enhancement in the antimicrobial activities of BO-ICs was shown against three different bacteria: *Escherichia coli*, *Staphylococcus aureus*, and *Pseudomonas aeruginosa*. BO-ICs displayed significant antibacterial activity against *Pseudomonas aeruginosa* with an MIC90 of 3.93 mg mL^−1^ and an MIC50 of 0.57 mg mL^−1^. Also, BO-ICs showed an increase in BO activity against *Escherichia coli* with an MIC95 of 3.97 mg mL^−1^, compared to free BO, which failed to show an MIC95. Additionally, BO-ICs showed a more significant activity against *Staphylococcus aureus* with an MIC95 of 3.92 mg mL^−1^. BO encapsulation showed significantly improved antimicrobial activities owing to the better stability, bioavailability, and penetration ability imparted by encapsulation into HPβCD.

## Abstract 2

*Boswellia sacra* essential oils, loaded in hydroxypropyl-beta-cyclodextrins, show promise as natural antimicrobials for controlling food microbial contamination.

## Graphical abstract

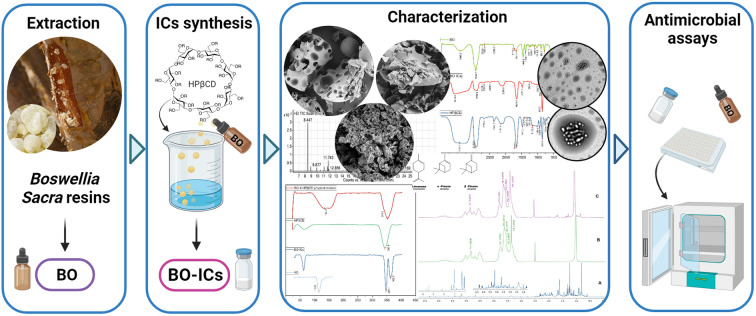

## 1. Introduction

Natural antimicrobials are gaining significant attention as promising substitutes to synthetic antimicrobials to address the rising risk of foodborne bacterial contamination and resistance.^1–3^ Essential oils (EOs), an enormous library of naturally occurring bioactive components with inherent antibacterial properties, are recognized as promising antimicrobial agents for managing of microbial infections and foodborne contamination.^1–3^

Several industries in the European Union employ EOs in several sectors, including food (additives/flavorings), pharmaceuticals, and perfumes. Also, individual compounds extracted from EOs were used in food flavorings.^4,5^ Besides their promising antibacterial and other therapeutic properties, most EOs are identified as *Generally Recognized as Safe* (GRAS), making them highly important and interesting as food additives and preservatives.^6,7^

Therefore, many commercial products utilize the antimicrobial activities of different EOs, such as using *Melaleuca alternifolia* EOs (extracted from tea tree oil) as antiseptics owing to their broad-spectrum antimicrobial activities.^8^ EOs of citrus, sage, and rosemary in the ‘DMC Base Natural’ product, commercially available in Spain, are used as food preservatives.^9^ Other EOs, not identified by the manufacturer, were used in the ‘Protecta One’ and ‘Protecta Two’ products commercially available in the US and used as food additives (identified as safe additives according to GRAS).^10^ Finally, carvone, isolated from the EOs obtained from Caraway seeds, is used in the ‘Talent’ product (The Netherlands) as a sprout inhibitor for potatoes and for fighting several fungal strains and diseases of stored potatoes.^11^

*Boswellia sacra* belongs to the Burseraceae family which mainly grows in Yemen, Oman, and Somalia.^12^ The resins of *B. sacra* have been utilized for several therapeutic purposes such as in chronic respiratory tract disorders, rheumatoid arthritis, malignant tumors, urinary tract disorders, and viral infections.^12,13^ The unique therapeutic properties of the *B. sacra* resins are mainly attributed to their EOs (BO), which were reported to have a rich content of different bioactive compounds such as polyphenols, terpenes, sesquiterpenes, and nonoxygenated terpenes.^14,15^ Furthermore, BO has shown remarkable anticancer and antiproliferative activities against various tumors such as breast, colon, and urothelial cancers.^13,15–18^ The BO safety was established on normal (non-cancerous) cells such as normal breast cells (MCF10-2A) and human embryonic kidney cells (HEK-293).^15^

Similar to other EOs, the therapeutic properties of BO are hindered by their low solubility and bioavailability, poor stability, and high volatility. Therefore, several studies have shown greater therapeutic efficacy of synthetic compounds and natural extracts upon their encapsulation into various carriers such as liposomes, macromolecules, polymeric nanoparticles, and chitosan nanoparticles.^15,19–22^ BO was encapsulated inside PLGA-PCL nanoparticles and showed greater antiproliferative and apoptotic efficiency against breast cancer.^15^

Cyclodextrins, a group of cyclic oligosaccharides, are composed of several glucopyranosyl units connected by α-(1,4) linkages. The most widely used cyclodextrins are α-, β-, and γ-cyclodextrins. The number of glucopyranose units incorporated in the chemical structures of cyclodextrins is used to differentiate them.^23,24^ Owing to their unique structures, composed of a hydrophilic surface and a hydrophobic cavity, cyclodextrins can host various molecules to develop their corresponding inclusion complexes (ICs) eventually. The formation of ICs with cyclodextrins is reported to improve the bioavailability, stability, release profile, physicochemical characteristics, and therapeutic efficacy of the encapsulated molecules.^24,25^ The attachments of hydroxypropyl functional groups to the surfaces of β-cyclodextrins can develop hydroxypropyl-beta-cyclodextrins (HPβCD), well-established with greater safety and solubility.^26^ Furthermore, the Food and Drug Administration (FDA) has approved using HPβCD as excipients in oral and intravenous solutions.^27,28^ Also, the FDA approved the use of α-cyclodextrins, β-cyclodextrins, and γ-cyclodextrins as food additives in 2000–2004 and were classified as safe (GRAS).^29,30^ In Europe, the use of β-cyclodextrins, in particular, was approved as a food additive (E 459) with an acceptable daily intake of 5 mg kg^−1^ day^−1^. The FDA's list of Inactive Pharmaceutical Ingredients includes sulfobutylether-β-cyclodextrins (SBE-βCDs) and HPβCD.^29,30^

Cyclodextrins can be found in a variety of pharmaceutical products due to their wide range of applications. For instance, they are employed in eye drops and aqueous parenteral solutions, nasal sprays, and tablets. In the European market, several medicinal uses of cyclodextrins and their derivatives are available, including the use of γ-cyclodextrins in Minoxidil solutions, β-cyclodextrins in Cisapride suppositories and Cetirizine tablets, randomly-methylated-β-cyclodextrins in nasal sprays (for hormonal replacement therapy), HPβCD in Itraconazole, and SBE-βCDs in parenteral antimycotic Voriconazole. Infusion preparations comprising alprostadil (prostaglandin E1) with α-cyclodextrins are also available in Japanese and German markets.^29,30^

Cyclodextrins encapsulated with natural extracts and EOs showed superior physicochemical and therapeutic properties such as enhanced bioavailability, stability, release sustainability, and antimicrobial activity of the encapsulated extracts.^31–36^ Numerous studies have documented the successful encapsulation of various EOs into HPβCD, demonstrating exceptional antibacterial efficacy. For example, encapsulating EOs of guava leaves improved their antibacterial activity against *Escherichia coli* (*E. coli*) by 2 fold and against *Staphylococcus aureus* (*S. aureus*) by 4 fold.^37^ Also, superior antimicrobial activity was evident upon encapsulating the EOs of yarrow, cinnamon, and lavender into HPβCD.^38–40^

Inspired by this context, this study sought the extraction of BO from *B. sacra* resins *via* hydrodistillation*,* formation of the ICs of BO with HPβCD (BO-ICs) by freeze-drying, characterization of BO-IC physicochemical properties, and the assessment of their antimicrobial activities against *Pseudomonas aeruginosa* (*P. aeruginosa*)*, E. coli*, and *S. aureus*. *S. aureus* and *E. coli* have been commonly associated with food contamination and food-borne pathogens,^41^ whereas *P. aeruginosa* could infrequently be linked to food illnesses although it represents an opportunistic bacterium that has been isolated from soil, vegetables, drinking water, and food resulting in serious illnesses.^42^ Also, targeting *P. aeruginosa* with conventional disinfectants and antiseptics is more challenging since it usually colonizes on common surfaces developing more resisting biofilms.^43^ Hence, in light of the FDA-approval of cyclodextrins use as food excipients and their safety profiles,^27–30^ BO encapsulation into HPβCD can enhance their antimicrobial activity and might further endorse their use as food preservatives and/or in food-packaging for controlling food contamination and food-borne pathogens.

## 2. Materials

### 2.1. Chemicals

The resins of *B. sacra* were obtained from their corresponding plants grown in Oman. (2-Hydroxypropyl)-beta-cyclodextrins (HPβCD) were supplied by Sigma (Sigma-Aldrich Co., Germany). KBr (FTIR grade) was provided by Merck (KGaA, Darmstadt, Germany). Nutrient broth was provided by Titan Biotech Ltd., Rajasthan, India. Acetonitrile (UV/HPLC grade) was purchased from VWR BDH® Chemicals (Fontenay-sous-Bois, France). Dimethyl sulfoxide (DMSO) was obtained from Fisher Scientific (Loughborough, UK). The rest of the chemical reagents were of analytical grade.

### 2.2. Microorganisms

The *P. aeruginosa, E. coli*, and *S. aureus* bacterial strains (ATCC numbers 27853, 25922, and 25923, respectively) were provided by Nawah Scientific Inc. (Cairo, Egypt). Bacteria were allowed to cultivate in nutrient broth and were incubated for 24 h at 37 °C. Bacteria were further maintained in glycerol (15% v/v), at −20 °C.

## 3. Methodology

### 3.1. BO extraction from the resins of *B. sacra*

BO was extracted from the resins of *B. sacra* using hydrodistillation.^15^ Briefly, the hydrodistillation process was carried out on the resins in an oven (Milestone ETHOS X, Milestone, Italy). The oven had been equipped with an IR temperature sensor and two magnetrons (950 W). The resins were first soaked in distilled water at a 1 : 5 (w/v) ratio, and the mixture was heated to 100 °C. The mixture could be blended well with the help of an electromechanical agitator. Other parameters were set at 2.3 bar for the pressure applied and 8 °C for the cooling system utilized, using a chiller (Smart H150-2100 chiller, LabTech, MA). The chiller was connected to a condenser to ensure a constant cooling temperature of 8 °C provided within the condenser. Several extraction cycles were delivered, where each cycle could be set at a specific time interval (45 min). Finally, the obtained extract at the end of each cycle was collected, and its corresponding volume was recorded.^15^

### 3.2. BO compositional analysis using GC-MS

BO composition was investigated using GC-MS analysis (Agilent Technologies gas chromatography (7890B) coupled with a mass spectrometer detector (5977B)), as previously reported.^15^

### 3.3. BO encapsulation into HPβCD

BO encapsulation into HPβCD was conducted by the freeze-drying technique to eventually develop their corresponding BO-ICs.^44,45^ Briefly, an aqueous solution of HPβCD was first prepared, adding 5 g of HPβCD to 25 mL of distilled water. Then, the prepared solution received 0.5 g of BO which was slowly added, and the final mixture was placed on a stirrer in the dark (200 rpm for 24 h, at 24 °C) to allow the development of BO-ICs. Consequently, the unencapsulated particles were removed from the obtained suspension by filtering it using 0.45 μm PTFE filters. As a result, the resulting solution, which now includes the BO-ICs only, was left in a freezer for 18 h, at −20 °C, before being lyophilized using a freeze-dryer for 72 h, allowing the sublimation of all the moisture contained (TOPT-10C freeze-dryer, Toption Group Co. Ltd., Xi'an, China). Finally, the lyophilized powders were kept in sealed containers inside a desiccator.

### 3.4. BO and BO-IC physicochemical characterization and bioassays

#### 3.4.1. Average particle size (Z-average) and polydispersity index (PDI) determination

Using a Zetasizer, the Z-average and PDI of BO-ICs and free HPβCD were determined (Nano-Zetasizer employing a dynamic light scattering DLS, Malvern Instruments Ltd, Malvern, UK). To accomplish this, BO-ICs and HPβCD were separately suspended in distilled water (w/v) (3 : 1 ratio) and samples were measured at room temperature.^46^

#### 3.4.2. Morphology of BO-ICs and free HPβCD

A Field Emission Scanning Electron Microscope (FESEM, LEO Supra 55, Zeiss Inc., Oberkochen, Germany) was used to investigate the morphological features of BO-ICs and HPβCD. Aluminum stubs were used to fixate the powder samples of BO-ICs and HPβCD, separately. Consequently, a thin layer of gold was sputtered (10 mA for 8 min) to coat the samples, before being observed at a magnification of 500×.^39^ Moreover, the encapsulation of BO and the morphology of BO-IC particles were examined using an Ultra-High Resolution Transmission Electron Microscopy (UHR-TEM), at 200 kV accelerating voltage (JEOL, JEM-2100 Plus, Tokyo, Japan). For this purpose, small amounts of BO-ICs and HPβCD were prepared in distilled water to form their corresponding suspensions. Samples were then sonicated (37 °C and 10 min). Afterwards, droplets of the final suspensions were positioned on copper grids, covered with a 1 nm carbon film, stained with uranyl acetate (2%), and left to dry using filter paper. Consequently, the samples were observed under 5k and 25k magnifications.^44^

#### 3.4.3. Fourier transform infrared spectroscopy (FTIR) analysis

Within a range of 4000–400 cm^−1^, the FTIR spectra of BO, HPβCD, and BO-ICs were obtained, using a Nicolet 380 FTIR (Thermo Scientific, Madison, WI). The spectra were examined for the presence of the characteristic peaks and the chemical structures of the recorded components and to ensure the BO encapsulation into HPβCD. BO-ICs and HPβCD were initially mixed with KBr (1 : 100) and then compressed into small discs utilizing a hydraulic press (15T manual press machine, China). For BO, a drop of oil was applied onto a piece of KBr and placed in front of an IR beam.^40^

#### 3.4.4. BO-IC encapsulation efficiency (% EE) and drug loading capacity (% DL)

Using a UV visible double beam spectrophotometer, the concentration of BO encapsulated into BO-ICs was determined spectrophotometrically at 254 nm (Cary 3500 UV-Vis Engine, Agilent Technologies, Pty Ltd, Mulgrave, Australia). Briefly, BO-ICs (5 mg) were suspended in acetonitrile (5 mL) and subsequently left in the dark at 24 °C, inside sealed containers while being mixed constantly for 72 h to facilitate the transfer of the BO entrapped to the solution. A calibration curve was developed for the BO, under the same conditions, in a range of concentrations of 3.125 to 200 μg mL^−1^ (BO: *y* = 0.0025*x* – 0.0053 and *r*^2^ = 0.9994). The % EE and % DL were then determined using the following eqn (1) and (2):^44^1
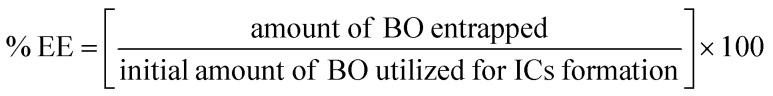
2
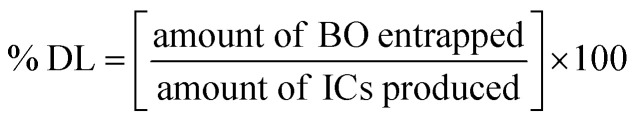


#### 3.4.5. H NMR spectra analyses

An NMR spectrometer (400 MHz, BRUKER BioSpin GmbH, D-76287 Rheinstetten, Germany) was used at 25 °C to obtain the ^1^H NMR spectra of HPβCD, BO, and BO-ICs. Also, the 2D-HNMR spectrum (NOESY) of the BO-ICs was obtained. Each sample was initially dissolved in DMSO.^31^

#### 3.4.6. Stability investigations

The thermal behaviors of HPβCD, BO, BO-ICs, and the physical mixture of BO and HPβCD (1 : 10) were investigated by analyzing their corresponding DSC curves obtained using a differential scanning calorimeter (model: DSC-60 plus, Shimadzu, Kyoto, Japan). Briefly, a constant rate of heat flow (10 °C min^−1^) was introduced, starting at 28 °C till reaching the end of the heat cycle at approximately 400 °C, under a nitrogen atmosphere.^44^

#### 3.4.7. Antimicrobial activity

HPβCD, BO, and BO-IC antimicrobial activities were examined against *E. coli*, *S. aureus*, and *P. aeruginosa* bacteria. For this purpose, direct broth microdilution assay was performed using 96-well plates, following previous studies with slight modifications.^31,47^ The bacteria were initially prepared, using 250 mL conical flasks, and then cultivated at 37 °C for 24 h. The obtained bacterial suspensions were diluted to 10^5^ CFU mL^−1^. For each bacterial strain, several aliquots (10 μL) of the suspension were added to two different 96-well plates; each well contained 90 μL of Nutrient Broth in addition to different concentrations of BO and BO-ICs. The concentrations of BO-ICs ranged from 0.25 to 4.09 mg mL^−1^. BO concentrations ranged from 0.34 to 5.4 mg mL^−1^. It is worth noting that the BO was added to the plates as an aqueous microemulsion, and their corresponding BO-ICs were added as an aqueous suspension following their filtration using syringe filters (0.20 μm). For negative control wells, only BO and BO-ICs were added to the culture media. For positive control wells, microbial suspensions were added to the culture media without BO or BO-ICs. The microplates were then incubated for 18 h at 37 °C after which the optical density was measured at 570 and 600 nm, utilizing a microtiter-plate reader (FLUOstar Omega, BMG Labtech, Ortenberg, Germany). Finally, the lowest concentration that showed no discernible microbial growth in the wells, following 24 h of incubation, was used to calculate the MIC values of BO and BO-ICs.^31,37,47–49^ Under the same conditions, a positive control of an antibiotic, ciprofloxacin, was examined within a range of concentrations of 10 to 0.001 μg mL^−1^ against the same strains of bacteria. Measurements were carried out as three replicates.

### 3.5. Statistical analysis

The mean ± standard deviation was calculated for each measurement. Three duplicates of each formulation were prepared. A *p* value of less than (or equal to) 0.05 revealed statistically significant differences. Also, one-way analysis of variances (ANOVA) was chosen to identify statistical differences.

## 4. Results and discussion

### 4.1. BO compositional analysis using GC-MS

**Fig. 1:**
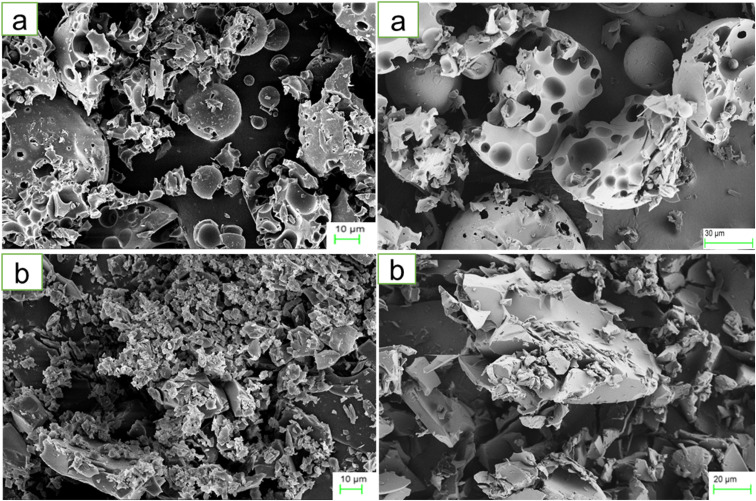
FE-SEM images of HPβCD (a) and BO-ICs (b). The HPβCD field exhibited varying-sized rectangular particles with some intact ovoid-shaped crystals, whereas the BO-ICs showed significant decrease and change in the sizes and shapes of the particles with many agglomerations. These alterations, shown by the BO-ICs, depict the successful formation of an amorphous product incorporating another compound in the complex, indicating the effective establishment of the inclusion complexes.

Thirty-two compounds were identified in BO using GC-MS analysis (ESI Table 1 and Fig. 1†).^15^ Comparing the obtained mass spectrum to the NIST library, the major compounds recognized were α-pinene (61.05%), d-limonene (9%), δ-3-carene (4.22%), camphene (3.67%), *O*-cymene (3.57%), and β-pinene (2.83%). These compounds belong to monoterpenes, whereas other chemical groups determined were oxygenated monoterpenes, sesquiterpenes, and esters. The GC-MS findings confirmed BO components' characteristics and identity and agreed with previous reports.^15,50^

### 4.2. Characterization of BO-ICs

The PDI and Z-average of HPβCD were 1.0 and 1.647 μm, respectively, reflecting a significantly polydisperse and heterogeneous system. The PDI index reflects the uniformity of particle sizes in a given dispersion. A system is considered extremely polydisperse with a PDI value higher than 0.7.^51^ The BO-IC suspension, on the other hand, demonstrated improved stability with considerably smaller and less scattered particles. The PDI and Z-average values determined for the BO-ICs were 0.1045 ± 0.0006 and 448.6 ± 0.6244 nm, respectively. The strong tendency of HPβCD and ICs to aggregate in water, as a result of the cyclodextrins inclination to self-assemble in water, can explain the above results.^44^ It is worth noting that the complexes formed by cyclodextrins and their hydrophilic aggregates have the ability to dissolve lipophilic substances *via* developing micelle-like structures and complexation.^52^ Similar results from earlier studies support the current findings.^39,52–54^

The morphology and size of HPβCD and BO-ICs were investigated using the FE-SEM images, presented in Fig. 1a and b, respectively. Along with some undamaged ovoid-shaped crystals, HPβCD exhibited varying-sized rectangular particles. Conversely, considerable morphological alterations in the crystal sizes and shapes of the BO-ICs were shown, and, more importantly, the BO-ICs showed a significant decrease in the crystal sizes compared to free HPβCD. Zetasizer analysis further evidenced the change in the particle sizes. In addition, many agglomerations were exhibited by the BO-ICs which can be explained by the increased tendency of the larger particles to gather and attract the smaller ones. The formation of the aggregates of the smaller particles and the changes observed in their morphologies suggest a complexation establishment, in which an amorphous product merged with an additional compound in the complex was developed. Similar findings were previously reported.^44,53^

**Fig. 2:**
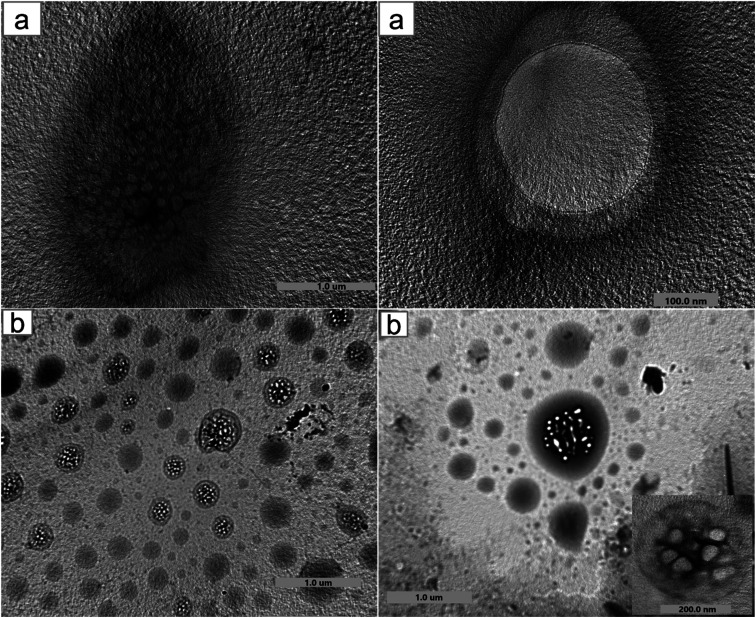
UHR-TEM images of HPβCD (a) and BO-ICs (b) show their structural and morphological characteristics and, in addition, BO encapsulation into HPβCD. Both samples showed round vesicles with thin layer membranes surrounding the particles. Also, several aggregates were shown by BO-ICs, and different shapes of micellar structures could be observed in both fields owing to the self-assembly of cyclodextrins.

Moreover, the encapsulation of BO and the structural and morphological characteristics of both HPβCD and BO-ICs were investigated using the UHR-TEM images obtained (Fig. 2). HPβCD particles (Fig. 2a) were presented with round shaped vesicles with diameters ranging between 82.7 and 334.2 nm and frequently developing larger micellar structures (self-assembled structures). On the other hand, BO-ICs (Fig. 2b) showed presumably spherical vesicles with a diameter ranging from 38.5 to 59.9 nm and a thin layer membrane surrounding the BO. Also, BO particles showed clear evidence of agglomeration in which larger particles attract smaller ones. These agglomerations were shown with diameters ranging from 136.9 to 336.8 nm. These findings refer to the positive establishment of BO-ICs and the successful encapsulation of BO into HPβCD. Nevertheless, the micellar structures revealed by both samples might be attributed to their preparations in distilled water, where cyclodextrins tend to self-assemble in water forming multiple agglomerates. Similar findings were reported supporting current results.^44,46,55,56^

### 4.3. FTIR analysis

**Fig. 3:**
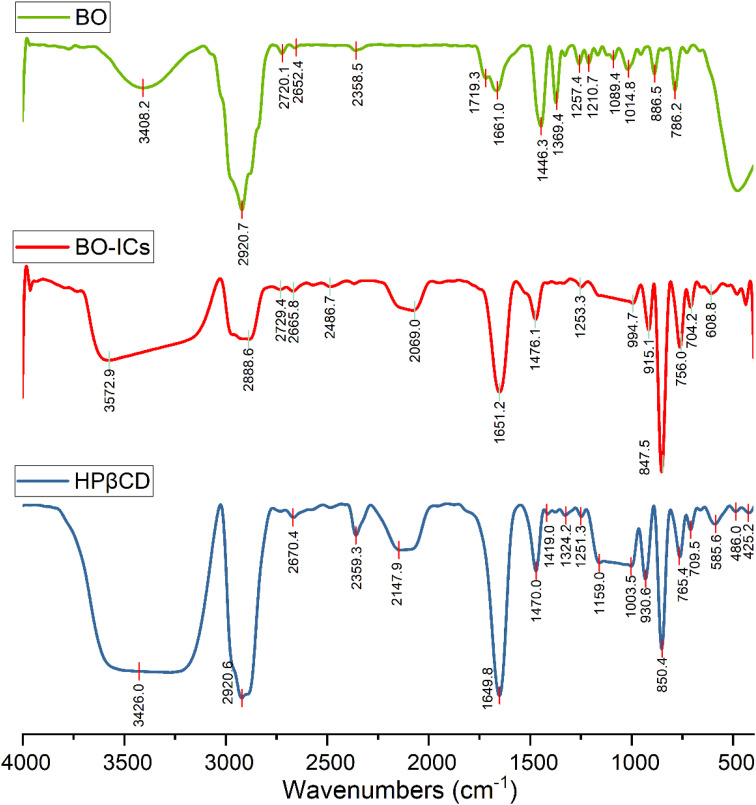
FTIR spectra of BO, BO-ICs, and HPβCD.

The encapsulation and complexation formation of BO-ICs were assessed by obtaining the FTIR spectra of HPβCD, BO, and BO-ICs (Fig. 3).

The FTIR spectrum of BO showed absorption bands at 3408.2 cm^−1^ (O–H stretching), 2920.7 cm^−1^ (methylene group stretching vibration), 2873.5 cm^−1^ (C–H stretching), 1661.0 cm^−1^ (H–O–H bending vibration), 1446.3 cm^−1^ (C–H scissoring vibration), 1369.4 cm^−1^ (C–O stretching vibration), 886.5 and 858.2 cm^−1^ (C–H bending of aromatic rings), 786.2 cm^−1^ (C–H bending), 1257.4 and 1089.4 cm^−1^ (C–O–C stretching vibration). Moreover, the FTIR spectrum of free HPβCD exhibited protruding bands of absorption at 3426.0 cm^−1^ (O–H stretching), 2920.6 cm^−1^ (methylene group stretching vibration), 2969.9 cm^−1^ (=CH_2_ symmetric stretching), 1649.8 cm^−1^ (H–O–H bending vibration), 1470.0 cm^−1^ (C–H vibration), and 1159.0 and 1003.5 cm^−1^ for the asymmetric and symmetric stretching vibrations of (C–O–C), respectively.

For the BO-IC FTIR spectrum, apart from the shifting and narrowing of the O–H bands as well as some other band shifts, all the BO absorption bands in the BO-IC FTIR spectrum were covered by the overlapping exerted by more intense bands of HPβCD. The encapsulation of some BO's compounds inside the HPβCD cavity and the possible interactions outside the HPβCD cavity may explain the prominent alteration presented in the O–H bands.^52^ The emergence of some inter-molecular hydrogen bonds between HPβCD and some BO components might indicate the above interactions.^40^ Furthermore, the broader band of the –CH_2_ chemical group, shown at 2920 cm^−1^, may indicate the successful entry of BO's lipophilic components into the HPβCD cavity.^40^ Consequently, these results show that BO was effectively encapsulated into HPβCD, making stable ICs.^31,39,40^ It is noteworthy that after six months, a re-examination of the corresponding FTIR spectra of BO and BO-ICs has been conducted to assess the stability of their primary functional groups and chemical constituents. Remarkably, identical spectra were acquired, indicating the stability of the chemical composition of BO and BO-ICs.

### 4.4. BO-ICs encapsulation efficiency (% EE) and drug loading capacity (% DL)

The calculated % EE and % DL of BO-ICs were 96.79 ± 1.17% and 10.87 ± 0.12%, respectively. The lengthy complexation allowed during the preparation procedure, whilst maintaining the ICs solutions well protected and firmly sealed throughout the preparation and drying processes, could explain the high encapsulation efficiency of BO-ICs. It was previously observed that the complexation time and drying processes significantly affect the proportion of the EOs encapsulated into HPβCD.^54^ Furthermore, several compounds detected in the BO, using the GC-MS analysis, have shown remarkable encapsulation affinities towards the HPβCD cavities, such as α-pinene, limonene, and δ-3-carene.^47^ These compounds, α-pinene, limonene, and δ-3-carene accounted for 61.05, 9.00, and 4.22% of the BO components, respectively. Hence, such intrinsic affinity supports the high % EE observed for the BO-ICs. Also, the BO-ICs FTIR analysis could further support these results. Moreover, the % DL of 10.87% ± 0.12% determined was reasonable, validating previous findings of similar ICs.^44^ For instance, the % EE and % DL of carvacrol into HPβCD, prepared using the freeze-drying method, reached 83.74 ± 1.15% and 8.25 ± 0.28%, respectively.^44^

### 4.5. H NMR spectra analyses

^1^H NMR was utilized to confirm the encapsulation and investigate the precise positioning of the BO components inside the hydrophobic cavity of HPβCD. Once BO (the guest molecule) approaches the inner side of the HPβCD's hydrophobic cavity, the proton atoms located at position 3 and position 5 (H3 and H5) at the HPβCD's inner cavity are chemically shifted.^57^

**Fig. 4:**
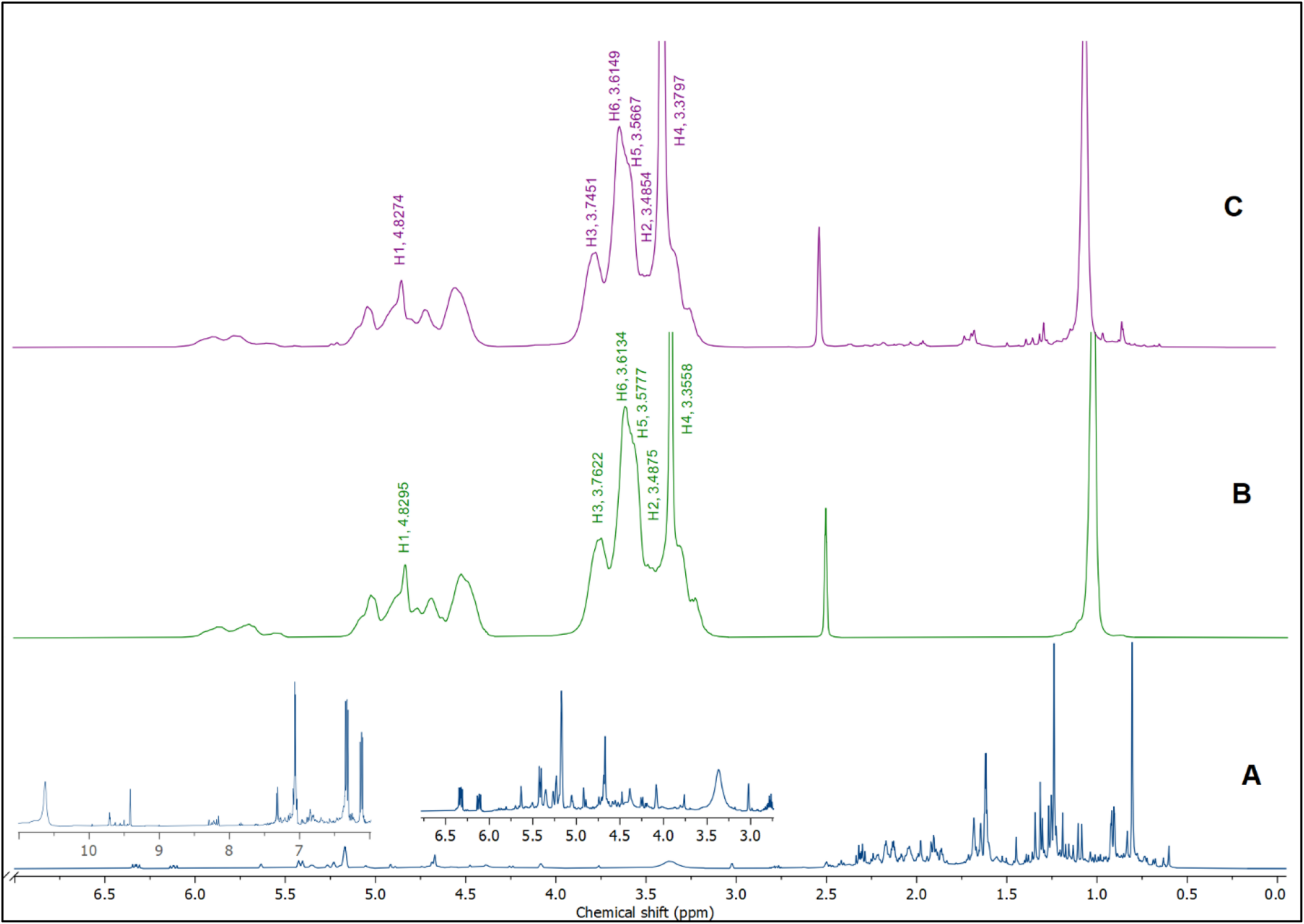
^1^H NMR spectra of BO (A), HPβCD (B), and BO-ICs (C).

The ^1^H NMR spectra of BO, HPβCD, and BO-ICs are shown in Fig. 4. BO's spectrum revealed recognizable proton peaks at 7.33, 7.07, and 6.85 ppm, confirming the presence of aromatic rings inside the molecular structure of some components present in the BO. The presence of double bonds was also indicated by the noticeable proton peaks appearing at 6.35 and 6.12 ppm. Protons shown at 3.76 and 3.37 ppm indicate that the molecular composition of BO has a C–O–C structure. Another characteristic proton peak of a methylene or a methyl structure linked to a double bond was observed at 1.86 ppm. These findings were in agreement with the analytical results obtained by the GC-MS analysis and FTIR.

Moreover, the BO-IC ^1^H NMR spectrum showed the proton peaks of both HPβCD and BO (Fig. 4). Additionally, Fig. 4B and C and Table 1 reveal the changes in the chemical shifts of the protons associated with the d-glucopyranose units of the HPβCD molecules. H1 and H2 were slightly shifted upfield (H1 is located in the middle structure of the hydrophobic cavity of HPβCD, whereas H2 is attached to the outside). Also, H6 was slightly shifted downfield (H6 is attached to the outermost side of the hydrophobic cavity). In contrast, H4 (located outside the cavity) showed a stronger shift downfield. Finally, H3 and H5 could also show stronger shifts upfield (H5 is located inside the depth of the HPβCD cavity, whereas H3 is closer to the wider edge). Based on these findings, the higher upfield shifts observed with H3 and H5 refer to the greater electron cloud densities developed around these protons. The greater electron cloud densities could have been induced by the stronger shielding exerted by the double bonds and the aromatic structures present in the BO, suggesting the effective development of the BO-ICs.

**Table 1:** Chemical shifts (*δ*) for HPβCD and BO-ICs and differences in chemical shift (Δ*δ*)

Proton no.	Free HPβCD (*δ*/ppm)	BO-ICs (*δ*/ppm)	Δ*δ* BO-ICs/free HPβCD (Δ*δ*/ppm)
H1	4.8295	4.8274	0.0021
H2	3.4875	3.4854	0.0021
H3	3.7622	3.7451	0.0171
H4	3.3558	3.3797	−0.0239
H5	3.5777	3.5667	0.011
H6	3.6134	3.6149	−0.0015

**Fig. 5:**
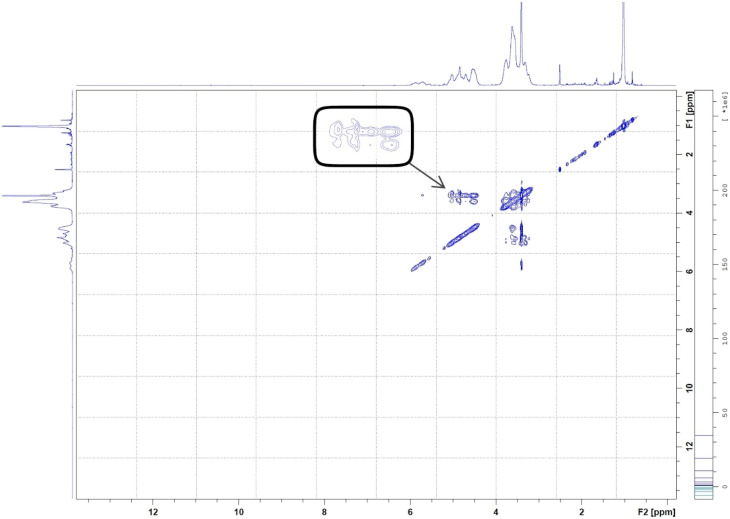
2D HNMR (NOESY) spectrum of BO-ICs. The spectrum showed the interaction between BO and HPβCD's protons.

On the other hand, 2D-HNMR NOESY (Nuclear Overhauser Effect Spectroscopy) was conducted on BO-ICs to investigate the inclusion mechanisms showing the entry of BO into the hydrophobic cavity of HPβCD. This method can detect possible cross correlations influenced by the spatial proximity that could develop among the BO components (guest) and HPβCD (host). The establishment of the BO-ICs, as shown by NMR spectroscopy, can be clarified most effectively by the Nuclear Overhauser Effect (NOE), which becomes more prominent by the spin polarization transfer, occurring between nearby atoms, moving from one population (HPβCD) to another (BO). Therefore, an interaction impacted by a couple of protons presented near each other, within 0.4 nm, in the space could be detected in a NOESY spectrum represented by the NOE cross correlation (crossed peaks).^58,59^ Hence, the obtained 2D HNMR NOESY spectrum of BO-ICs (Fig. 5) could reveal a substantial spatial proximity and correlation, between BO protons and HPβCD protons (H3 and H5). This correlation supports the chemical shifts revealed in the ^1^H NMR spectra and hence denotes the successful encapsulation of BO into the HPβCD cavity while effectively forming their BO-ICs. Similar results were reported previously showing the effective encapsulation of various molecules into HPβCD cavities while revealing similar strong correlations as could be shown by 2D NMR spectroscopy analyses.^60,61^ It is worth noting that the 2D-HNMR NOESY analysis was conducted five months after the initial preparation of BO-ICs, supporting their high stability profile.

### 4.6. Stability investigations

BO's physical characteristics were closely monitored over a period of 18 months following their extraction, including BO's color, viscosity, and aroma, while being kept in a sealed container in the dark at 4 °C. Interestingly, these characteristics have not changed over time, referring to the high stability of the extracted BO.

Furthermore, in order to confirm the positive establishment and investigate the thermal stability and behavior of the BO-ICs, the DSC curves of HPβCD, BO, BO-ICs, and a BO-HPβCD physical mixture were obtained. Briefly, the disappearance or shifting of the endothermic melting peak of BO (the guest molecule) on the DSC thermogram of BO-ICs served as evidence of the successful formation of BO-ICs and effective encapsulation of BO into HPβCD.^44,62^

**Fig. 6:**
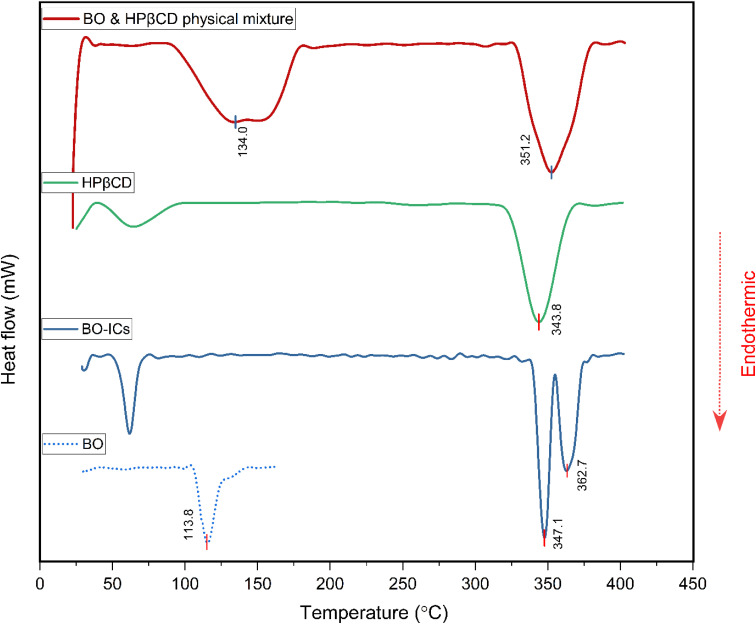
DSC thermograms of BO, BO-ICs, HPβCD, and the BO-HPβCD physical mixture.

The DSC thermogram of HPβCD (Fig. 6) revealed two endothermic peaks. The first peak appeared below 100 °C, indicating the vaporization of water molecules, whereas the second peak was shown at 343.8 °C, depicting the HPβCD melting. Furthermore, BO's DSC thermogram showed a sharp endothermic peak at 113.8 °C associated with the BO boiling point.

The DSC thermogram of the BO-ICs exhibited one endothermic peak below 100 °C, associated with the water molecules vaporization, in addition to two sharp endothermic peaks at 347.1 and 362.7 °C indicating the successive processes of melting of HPβCD followed by boiling of the released BO, respectively. Furthermore, the DSC thermogram obtained for the physical mixture of HPβCD and BO showed the melting peaks of HPβCD and BO at 351.2 and 134.0 °C, respectively. Hence, the disappearance of the endothermic peaks shown at 113.8 °C and at 134.0 °C in the BO and in the physical mixture's thermograms, respectively, from the BO-ICs thermogram refers to the successful encapsulation of the BO into HPβCD and the formation of BO-ICs. Therefore, investigating the BO-ICs thermogram might depict the following incidents associated with the thermal decomposition process. First, the relatively thermostable structure of HPβCD started to melt at around 330 °C, resulting in the release of the thermolabile components of BO and their rapid decomposition. These results verify the successful formation of the BO-ICs and superior thermal stability compared to free BO. Similar observations were previously reported.^44,45,62,63^ It is important to note that the DSC analysis of the BO-HPβCD physical mixture was carried out four months after the initial preparation of BO-ICs. This may also support the high stability profile of BO, where the same BO boiling points were observed.

The non-hygroscopic, homogeneous, and crystalline structure of HPβCD coupled with a hydrophobic cavity and a hydrophilic surface can explain the greater stability profiles developed upon their use to encapsulate other molecules, forming larger micellar/self-assembled structures known with superior characteristics (as revealed by both the UHR-TEM and FE-SEM analyses herein), encompassing molecules of various properties and polarities.^64,65^ These features further depict the significant stability, therapeutic properties, and bioavailability of the encapsulated molecules. In addition to the molecules' encapsulation into the hydrophobic cavities of HPβCD, the possibility of other interactions on the surfaces of the HPβCD particles greatly supports the stable profiles of the developed complexes as supported by stability and morphological findings.^64,65^ In fact, previous reports showed that some drugs formed covalent linkages with the OH functional groups furnished by the cyclodextrins, increasing their stability, targeting ability, and sustained release of their cargo.^65^

### 4.7. Antimicrobial activity investigation

The antibacterial activities of HPβCD, BO, BO-ICs, and ciprofloxacin were assessed against *S. aureus*, *P. aeruginosa*, and *E. coli* bacteria, and the obtained MIC values are shown in Table 2.

**Table 2:** MIC values of free BO, BO-ICs, and ciprofloxacin. Spline-LOWESS was used to determine the significant MIC values (*p* < 0.05) within the range of concentrations examined, 0.34 to 5.4 mg mL^−1^ for free BO, 0.25 to 4.09 mg mL^−1^ for BO-ICs, and 10 to 0.001 μg mL^−1^ for ciprofloxacin

	*P. aeruginosa*			*E. coli*			*S. aureus*		
	MIC95	MIC90	MIC50	MIC95	MIC90	MIC50	MIC95	MIC90	MIC50
BO	>5.4 mg mL^−1^	>5.4 mg mL^−1^	>5.4 mg mL^−1^	>5.4 mg mL^−1^	2.85 mg mL^−1^	1.15 mg mL^−1^	>5.4 mg mL^−1^	4.94 mg mL^−1^	1.13 mg mL^−1^
BO-ICs^a^	>4.09 mg mL^−1^	3.93 mg mL^−1^	0.57 mg mL^−1^	3.97 mg mL^−1^	2.38 mg mL^−1^	<0.25 mg mL^−1^	3.92 mg mL^−1^	3.32 mg mL^−1^	<0.25 mg mL^−1^
Ciprofloxacin	0.29 μg mL^−1^	0.26 μg mL^−1^	0.11 μg mL^−1^	0.16 μg mL^−1^	0.14 μg mL^−1^	0.02 μg mL^−1^	0.1 μg mL^−1^	0.09 μg mL^−1^	0.05 μg mL^−1^

a Values were determined based on the encapsulation efficiency.

The abundance of monoterpenes and oxygenated monoterpene compounds in BO might be attributed to the potent antibacterial properties of BO. These compounds include, but are not limited to, α-pinene, limonene, β-pinene, thymol, and carvacrol, which have shown excellent targeting ability towards the microbial cell membranes enhancing their permeability.^39,66–68^ Additionally, the integration and disruption of the lipid bilayers of microbial cells would make them more vulnerable to being targeted by terpenoid compounds and their analogs, owing to their lipophilic nature. These properties enhance the permeability of the microbial cell membranes, impairing the essential cellular transport mechanisms leading to bacterial death.^66^

Ciprofloxacin is a potent antibiotic which exerts a broad spectrum of activity against both Gram-positive and Gram-negative bacteria.^69^

Free HPβCD could not show any antibacterial activities (>100 mg mL^−1^) against the microorganisms tested, suggesting the absence of possible synergistic effects imparted by HPβCD particles. Also, free BO failed to reveal any antibacterial activity against *P. aeruginosa* within the range of concentrations examined. However, BO-ICs showed significant antibacterial activity with an MIC90 of 3.93 mg mL^−1^ and an MIC50 of 0.57 mg mL^−1^. Also, BO-ICs showed an increase in BO activity against *E. coli* with an MIC95 of 3.97 mg mL^−1^, compared to free BO which failed to reveal an MIC95 value within the range of concentrations tested. Additionally, BO-ICs depicted a more significant activity of BO against *S. aureus* with an MIC95 of 3.92 mg mL^−1^, compared to free BO which failed to show an MIC95 value within the range of concentrations examined.

These findings explicitly demonstrate the significance of encapsulating BO into HPβCD, improving their penetration capability, solubility, stability, and ultimately their antibacterial activity at lower BO concentrations. Since EOs are well-established for exerting their antibacterial activity at the bacterial cell membranes and in the bacterial cytoplasm, the potential increase of the BO aqueous solubility upon their encapsulation into HPβCD may have improved their targeting of the active sites of bacteria.^46,70^ It has been documented that limonene, one of the main components detected in BO by GC-MS (ESI Fig. 1 and Table 1†), exerts its antibacterial activity against *E. coli* by increasing the bacteria membrane permeability, as could be shown by Attenuated Total Reflectance Infrared Micro-Spectroscopy.^71^ Also, α-pinene, the major component in BO, limonene, and β-pinene (ESI Fig. 1 and Table 1†) could exert a significant inhibition of the respiratory activities of the mitochondria, either isolated or in yeast cells.^72–74^ Nevertheless, these active components in addition to others found in BO have been reported to face several challenges, like other EOs, where they have poor liquid solubility, limiting their diffusion in biological fluids, coupled with the intrinsic nature of the high volatility of EOs, negatively impacting their stability in similar fluids.^70^ These two major issues facing BO could be addressed by encapsulation into HPβCD, owing to the positive impacts of similar encapsulations in improving solubility and imparting release sustainability of their cargos.^39,46,70–72^ In summary, HPβCD, as an acclaimed drug delivery carrier, facilitated the access of BO's components to their active sites in the targeted bacteria while protecting their cargo from degradation/elimination and controlling their release, augmenting their stability, targeting ability, and therapeutic efficacy.

## 5. Conclusions

EOs derived from different plants have exhibited favorable antimicrobial, antitumor, and antioxidant properties. BO has demonstrated outstanding therapeutic properties mainly attributed to its abundant content of terpenoids and other bioactive phytochemicals. In this study, BO was extracted from *B. sacra* resins *via* hydrodistillation and then encapsulated into HPβCD by freeze-drying. The obtained BO-ICs exhibited high entrapment efficiency, stability, and antimicrobial activities. The BO-ICs formation and stability were successfully verified using DSC, FTIR, FE-SEM, UHR-TEM, ^1^H NMR, and 2D HNMR NOESY analyses. Ultimately, as compared to free BO, significant improvement in the antibacterial activities of BO-ICs was demonstrated against *E. coli*, *S. aureus*, and *P. aeruginosa*, supporting the advantageous impacts of BO encapsulation into HPβCD to achieve high stability, bioavailability, penetration ability, and therapeutic effects. Therefore, these findings, further augmented by the established safety profiles of BO and HPβCD, support the promising utility of BO-ICs for controlling bacterial growth in food and for other cosmetic and therapeutic uses.

## Conflicts of interest

The authors declare no competing interests.

## Supplementary Material

NA-006-D3NA00882G-s001
