# Supplementary material for: Improved antimicrobial activities of Boswellia sacra essential oils nanoencapsulated into hydroxypropyl-beta-cyclodextrins
Source: Nanoscale Adv. 2023 Dec 22;6(3):910–24. doi: 10.1039/d3na00882g (PMC10825941; doi:10.1039/d3na00882g)
Supplement: NA-006-D3NA00882G-s001 [file NA-006-D3NA00882G-s001.pdf]

Electronic Supplementary Material (ESI) for Nanoscale Advances.
This journal is © The Royal Society of Chemistry 2023

Improved Antimicrobial Activities of Boswellia Sacra Essential Oils Nanoencapsulated into Hydroxypropyl-beta-cyclodextrins

Obaydah Abd Alkader Alabrahim[1], Salim Alwahibi[2], and Hassan Mohamed El-Said Azzazy[1,3*]

[1]Department of Chemistry, School of Sciences & Engineering, The American University in Cairo, New Cairo 11835, Egypt. Obaydah.alabrahim@aucegypt.edu; hazzazy@aucegypt.edu

[2]Falha Medical Solutions, Muscat 113, Oman.

[3]Department of Nanobiophotonics, Leibniz Institute of Photonic Technology, Jena, Germany.

Corresponding authors:

Prof. Hassan M. E. Azzazy

School of Sciences & Engineering

The American University in Cairo

AUC Avenue, SSE # 1184, P.O. Box 74

New Cairo, Egypt 11835

E-mail: hazzazy@aucegypt.edu

Office: +2 02 2615 2559 (GMT+2 hr)

Supplementary Figure 1.

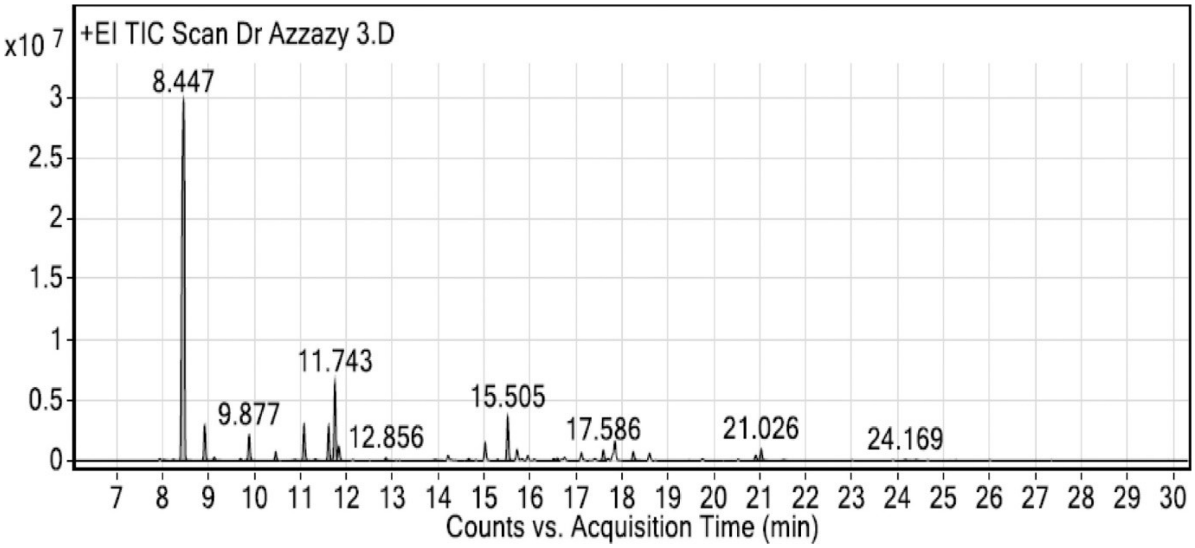

Figure 1. GC-MS chromatogram of BO.[1] Reproduced from ref (1). Copyright 2022 American Chemical Society.

Supplementary Table 1.

Table 1. BO's Chemical Composition.[1] Reproduced from ref (1). Copyright 2022 American Chemical Society.

Peak no.	RT	Compound name	%
		**Monoterpenes**	
1	8.024	tricyclene	0.11
2	8.229	α-thujene	0.13
3	8.447	α-pinene	61.05
4	8.908	camphene	3.67
5	9.118	verbenene	0.43
6	9.69	*p*-cymene	0.25
7	9.798	sabinene	0.09
8	9.877	β-pinene	2.83
9	10.454	β-myrcene	0.94
10	10.873	α-phellandrene	0.16
11	11.073	δ-3-carene	4.22
12	11.189	*p*-cymenene	0.06
13	11.32	α-terpinene	0.19
14	11.608	*o*-cymene	3.57
15	11.743	d-limonene	9
17	12.856	γ-terpinene	0.35
24	17.586	alloocimene	1.35
29	21.52	*R*(+)-limonen	0.11
		total	88.51
		**Oxygenated Monoterpenes**	
16	11.827	eucalyptol	1.7

Peak no.	RT	Compound name	%
18	13.922	*trans*-d-dihydrocarveol	0.22
20	15.286	6-camphenol	0.19
21	15.505	2,3-epoxycarane, (*E*)-	4.86
22	16.506	verbenol	0.24
23	16.585	pinocarvone	0.33
25	18.238	verbenone	1.18
26	19.453	carvone	0.06
		total	8.78
		**Esters**	
27	20.905	bornyl acetate	0.66
28	21.026	bicyclo[2.2.1]heptane-3-methylene-2,2-dimethyl-5-ol acetate	1.44
		total	2.1
		**Sesquiterpenes**	
30	24.169	β-bourbonene	0.17
31	24.401	β-elemene	0.11
32	25.267	caryophyllene	0.07
		total	0.35
		**Others**	
19	14.658	6-isopropenyl-3-methoxymethoxy-3-methyl-cyclohexene	0.25

References:

1. Azzazy, H. M. E.-S.; Abdelnaser, A.; Al Mulla, H.; Sawy, A. M.; Shamma, S. N.; Elhusseiny, M.; Alwahibi, S.; Mahdy, N. K.; Fahmy, S. A., Essential Oils Extracted from Boswellia sacra Oleo Gum Resin Loaded into PLGA–PCL Nanoparticles: Enhanced Cytotoxic and Apoptotic Effects against Breast Cancer Cells. *ACS Omega* **2023,** *8* (1), 1017-1025 DOI: 10.1021/acsomega.2c06390.
